# Revealing a gap in the clinical competence of nursing staff in nursing homes: a cross-sectional study with the Ms. Olsen test

**DOI:** 10.1186/s12912-023-01297-7

**Published:** 2023-04-18

**Authors:** Irén Vikström-Dahl, Pia Cecile Bing-Jonsson, Auvo Rauhala, Lisbeth Fagerström

**Affiliations:** 1 Faculty of Education and Welfare Studies, Åbo Akademi University, Vaasa, Finland; 2 Faculty of Health and Social Sciences, University of South-Eastern Norway (USN), Kongsberg, Norway; 3 Finnish Centre for Client and Patient Safety, Wellbeing Services County of Ostrobothnia, Vaasa, Finland; 4 Faculty of Health and Welfare, Novia University of Applied Sciences, Vaasa, Finland

**Keywords:** Clinical competence, Nursing homes, Geriatric nursing, Clinical decision-making, Geriatric assessment, Nursing assessment, Finland, Translation, Licensed practical nurse, Registered nurse.

## Abstract

**Background:**

Nursing staff, caring for frail older people in long-term care, needs to have a certain level of clinical competence to identify diseases at an early stage and to assess and provide good nursing care. In Finland, nursing care is based on evidence-based and high-quality nursing care. However, earlier inspections by the National Supervisory Authority for Welfare and Health showed many discrepancies between the nursing staff’s clinical competence and adequate and continuous education.

**Aim:**

This study aimed to explore nursing staff, i.e. the clinical competence and decision-making skills of registered and practical nurses in nursing homes for older people in Finland and to analyse the association between nurses’ clinical competence and fundamental background factors.

**Methods:**

We conducted a cross-sectional study of 337 participants in 50 nursing homes in the western part of Finland, between December 2020 and January 2021. The instrument used was the validated Ms. Olsen test, an extraction of NOP-CET. Statistical analyses were conducted with descriptive statistics and correlations and a cut-off for clinical competence.

**Results:**

This study with the Ms. Olsen test revealed that only one-fourth of the RNs and a third of the PNs passed the clinical competence test. In the self-evaluation, almost all participants evaluated themselves with *good* clinical competence. The Finnish Current Care Guidelines were used on a daily basis by 7.4% and weekly by 30%. Significant correlation was found between Swedish as a working language and mother tongue and the score for clinical competence.

**Conclusion:**

The clinical competence test, the Ms. Olsen test, was used in Finland for the first time to evaluate the nursing staffs´ clinical competence in nursing homes. We found gaps in the clinical competence in Finnish nursing homes, both for PNs and RNs. The result differed remarkably from their self-assessments and the staff did not use the national nursing guidelines as required to develop their nursing skills and knowledge. Gaps in the clinical competence have been identified and can be used to develop targeted continuous education.

## Background

Worldwide, the older population is increasing [1], and life expectancy has improved by six years up to 73.3 years between 2000–2019 [2]. Long-term care (LTC) for older people includes a wide range of nursing care for older people with limited capability for self-care because of chronic diseases [3]. In Finland, these services include both nursing care and non-health-related care to support optimal quality of life and daily activities [4]. These patients with age-related diseases and multiple health-challenges presuppose advanced nursing care performed by nursing staff with high clinical competence. During the last few years, the media in Finland has repeatedly reported complaints and shortcomings in the LTC, despite the fact that Finland’s high level of education in nursing is well known.

Competence is often related to professionals’ performance in the nursing field. The International Council of Nursing defines competence as the “effective application of a combination of knowledge, skills and judgement demonstrated by an individual in daily practice or job performance*”* [5]. Competence is more than just performance; it is nursing knowledge, skills, personal capacity and ability to judge and make decisions and to evaluate a patient’s wellbeing and is reflected by the nurses’ individual attributes and attitudes [5–7]. Fagerström [8] describes clinical competence inspired by Aristotle’s episteme, techne and phronesis, such as evidence-based knowledge, skills and wisdom/ethics. Benner [9] describes five levels of nursing competence: novice, advanced beginner, competent, proficient and expert, meaning the more working years nurses have, the more competence will be shown. To maintain competence, nurses need to obtain relevant continuing education [10]. Nurses working between 5 and 10 years have been shown to reach a plateau in their competence level, meaning that there is no correlation between years of working and level of competence. Hence, some nurses were striving for more competence, whereas others did not [11, 12].

Attitudes and beliefs, interaction, evidence-based care, educative competence, leadership and competence in development were identified as important factors for nurses in elderly care, from the perspective of registered nurses (RN), practical nurses (PN), clinicians and nursing leaders [13]. Important clinical competence factors include planning, assessment and making independent decisions for patients’ nursing care. A nurse needs to detect changes in older patients’ health and situations and to have the capacity to act immediately [13, 14]. For registered nurses (RN) and practical nurses (PN), clinical competence includes health promotion, risk management, supporting patient resources and rehabilitation, basic care, acute and chronic diseases, knowledge of geriatric and general long-term illnesses, behavioural changes (memory disorders), managing challenging situations, practical nursing tasks, pain management, medication competence and evaluation and end-of-life care [15, 16]. According to the same review the Finnish study [17] showed that close relatives have high expectations of the clinical competence of nurses in LTC homes to recognise symptoms of diseases and to have knowledge about patients’ individual needs. Nurses who have continued with their education (with extended education) are considered to develop nursing care and to influence other staff positively through guidance and coaching [18]. The clinical competence of the nursing staff in Finland has only been evaluated earlier with self-evaluated instruments [19, 20]; no objective test of their clinical competence has been done.

The regulations of nursing care in Finland are based on evidence, good nursing practices and routines and high quality. Safe and formally competent staff are needed [21–23] and the Current Care Guidelines are included in the Terveysportti database, which is published by the Finnish Medical Society Duodecim [24]. The National Supervisory Authority for Welfare and Health [25] supervises LTC for older people, and during their inspection visits, many inconsistencies have emerged regarding a lack of staff. Drug treatments have had a negative impact on patient safety by not checking the clinical competence of nursing staff. In addition, there were shortcomings in management, accountable people were missing in departments, and adequate education and competence were lacking.

Therefore, the study aimed to explore nursing staff, i.e. the clinical competence and decision-making skills of registered and practical nurses in nursing homes for older people in Finland and to analyse the association between nurses’ clinical competence and fundamental background factors.

## Method

### Long-term care for the older people in Finland

The LTC for the older people in Finland is arranged in home care, sheltered housing or institutional care. In sheltered housing, patients have their own apartments with social and healthcare service/assistance available round the clock. When patients cannot live in their own home or in shelter housing for medical reasons or patient safety, institutional care is arranged in nursing homes or wards in health centres with 24-hour care. The need for assistance is according to patients’ needs and is organised by the municipalities or can be arranged by third (non-profit) and private (for-profit) sectors. The municipality can buy the service from the private sector; the patients pay the total fee but are partly compensated by the municipality, depending on their income level. Nursing staff are RNs, who are often available during the daytime, and PNs, who work around the clock [26]. When this research was done, the patient-to-nurse ratio was 0.5 per day, according to Finnish law, including all nursing staff who are in close contact with patients. From April 2023, the ratio is expected to be 0.7 per day [27]. Employers are responsible for guaranteeing that the nursing staff have the necessary competence to care for patients; the only specific competence mentioned is related to drug administration [28].

Finland is a bilingual country, with both Finnish and Swedish as the official national languages [29]. In 2020, 5.2% of the population was Swedish-speaking and 87% spoke Finnish [30]. Patients have the right to receive good treatment and nursing care in their own mother tongue and nursing staff need to manage both languages [22, 31].

Quality recommendations by Ministry of Social Affairs and Health [32] clarify that nursing staff need to have a certain level of clinical competence to identify dementia diseases at an early stage and to assess and provide good nursing care to patients with different diseases. Nursing staff need to receive continuous education in safe drug administration and pain relief, palliative care, and end-of-life care. The level of their clinical competence needs to be followed up on and improved.

The majority of the nursing staff are PNs taking care of patients 24 h a day. An RN is in charge in the daytime and a physician is available round the clock and will visit the nursing home for up to four times a month, depending on the level and needs of the patients [33].

### Description of the development of the NOP-CET to the Ms. Olsen test

The Ms. Olsen test was developed to measure the clinical competence and decision-making of nursing staff [34]. The Ms. Olsen test was developed from the Nursing Older People-Competence Evaluation Tool (NOP-CET), established in 2013. The NOP-CET is a long-sheet instrument and the Ms. Olsen test was extracted from it [34, 35].

The NOP-CET is a questionnaire with a focus on measuring nursing staff’s clinical competence in elderly care, both in home care and institutional care (nursing homes). This self-evaluating questionnaire was developed in Norway over a 2-year period (2012–2013). It consists of 65 domains with several items and a total of 346 sub-items. The scaling in the questionnaire consists of both Likert-type scales and dichotomous scores (meaning true/false, correct/incorrect). The participants of the NOP-CET were nursing staff in elderly and home care, such as practical nurses, nurses, managers, ordinary staff members and students working on a time basis with shorter or longer contracts [6]. The validation of the NOP-CET is based on classical test theory and has shown good content and construct validity, reliability, precision, interpretability, acceptability and feasibility [35].

Feedback has revealed that the instrument was too long and time-consuming; however, nurse managers were interested in healthcare personal’s clinical decision-making skills. The review of the NOP-CET showed that the fictious patient Ms. Olsen test included in the questionnaire was relevant to the nurse managers’ *opinions*. The Ms. Olsen test case includes five categories relevant for clinical decision-making skills: “treatment”, “assessment and taking action”, “cover basic needs”, “responsibility and activeness” and “cooperation”. The Ms. Olsen test is a 19-item questionnaire with statements on clinical competence and decision-making skills. It is not a shorter version of NOP-CET but an extraction from the NOP-CET that measures clinical decision-making skills [34, 35].

To obtain reliable cut-off points showing clinical competence and decision-making skills for the Ms. Olsen test, additional analyses were needed. An analysis based on Rasch measurement theory (RMT) was performed for RNs and PNs. For RNs, the RMT showed that they needed to get a score of 66% correct to achieve clinical competence; PNs needed to have half (50%) of the items correct to achieve clinical competence [34].

### Translation process

The Ms. Olsen test was available in English and Norwegian. As Finland is a bilingual country, it needed to be translated into Finnish and Swedish. The translation process followed the accepted and standardised translation guidelines of the Mapi Institute in Lyon, France [36–38]. Professional translators did the first forward translation from English to Swedish and Finnish. Bilingual speakers did the second and back translations from Swedish and Finnish to Norwegian and English. An expert group, including RNs with Swedish or Finnish as their mother tongue and the developer of the instrument, reviewed the translations and focussed on cultural sensitivity and nursing terminology. Even though the Norwegian language is close to Swedish, finding the right nursing terminology was challenging. The correct accent needed to be found in the Swedish and Finnish languages. Latin words are commonly used in nursing care in Finland because of the bilingual nation and working environment [36–38]. A pilot group including both Swedish- and Finnish-speaking nurses performed quality control, giving feedback about the translations, cultural differences and terminology, both in Swedish and Finnish. After discussions with the proofreading pilot group and consensus was reached, the final version of the instrument was approved [36]. In this study the instrument is still under development in Finland and will be tested for validity, reliability and cultural adaptation in the future.

### Aim

The aim of this study was to explore RNs’ and PNs’ clinical competence and decision-making skills in nursing homes for older people in Finland and to analyse the association between nurses’ clinical competence and central background factors. The instrument used for this research was the Ms. Olsen test. Our research questions were as follows:

- What is the level of clinical competence and decision-making skills of RNs and PNs?
- What central background factors are associated with clinical competence and decision-making skills for RNs and PNs?

### Design and setting

An explorative cross-sectional study design was used, covering long-term care in nursing homes for the older people in western Finland.

### Instrument

The validated instrument used was “the Ms. Olsen test” [34]. The instrument is a short test to assess the participants’ clinical competence and decision-making. The Ms. Olsen test has a fictitious patient, Ms. Olsen, a 90-year-old woman who suffers from normal weaknesses from old age. The questionnaire is described in Table 1.

**Table 1:** Description of Ms. Olsen test and number of correct answers per item for RNs respectively PNs *Ms. Olsen is 90 years old and generally weakened by age. Imagine that she develops the following symptoms* *Please choose how you would respond when Ms. Olsen, your patient develops the following symptoms. You may choose up to two options on each line*

Item	Item wording statement	Number of correct answers RN	Number of correct answers PN
1	Has dyspnoea during rest within last two days	1	2
2	Choughs, has increased saliva and respiration frequency above 20/min	2	1
3	Has temperature above 38.5	2	1
4	Is substantially dehydrated	2	1
5	Skin has rash, wounds, is red or itchy	1	1
6	Has reduced appetite and food intake	2	2
7	Is not able to eat	2	1
8	Has pain and discomfort in mouth	1	1
9	Is incontinent for urine, stings when urinates	2	1
10	Has fresh blood in stool	1	2
11	Has increased needs to full care within last two days	2	1
12	Has fallen two times during previous week	2	1
13	Has symptoms of partial paralysis	1	2
14	Is more tired during the day	1	1
15	Has changes in sight, hearing, speech and comprehension	1	2
16	Has newly occurring chest pain	1	2
17	Has lost interest in keeping home in order, sleeps in chair instead of bed	1	1
18	Has short attention span and delusions	1	2

Participants need to imagine that Ms. Olsen will develop 19 different symptoms and decide and act according to six response categories: (1) *no action required*, (2) *observe following day*, (3) *consult an RN*, (4) *nursing-related actions immediately*, (5) *assessed by a physician* and (6) *acute help required*. Participants can choose a maximum of two categories for each symptom. All the symptoms require actions from the nursing staff, meaning category 1–2 is never correct. Depending on the severity of the symptoms and the participants’ education and profession, 3–6 is correct. Category 3 can only be correct for PNs consulting an RN, and 4–6 is the correct answer for RNs. The score sheet for Finland was checked according to Finnish Current Care Guidelines [24] and with an expert group, including a physician, two nursing teachers and two RNs working in elderly care. The score sheets in Finland and Norway are similar [34]. The correct score sheet is different for RNs and PNs because RNs are expected to undertake independent examinations, notify a doctor or call for emergency assistance, depending on the seriousness of the situation and education [15, 16, 23]. In the Ms. Olsen test, 18 of the 19 items in the questionnaire were used, and one item was removed, due to an error in the participants´ interpretation of the item, which was contradictory.

The introduction to the questionnaire included information regarding the participants’ confidentiality and ethical considerations to participate in the study. In addition to the Ms. Olsen test, the questionnaire also comprised demographic information, such as gender, age, education, language, employment status, work experience and continuous education. The questionnaire included statements on how often the nursing staff were seeking evidence-based information and national guidelines and the scale was: *never, every month, every week, every day* and *not relevant*. In addition, the nursing staff were asked to self-evaluate their own clinical competence, with a scale ranging from very weak to very good.

### Participants and data collection

The participants of this study were all RNs and PNs in nursing homes (both public and private) in two regions in the western part of Finland. The RNs’ education is on bachelor level while the PNs’ are on vocational level. In Finland, both RNs and PNs are expected to assess the patients’ health status as presented in the test. Their level of response, and thus what is scored as correct/wrong on the test, is different between the two groups and in accordance with their educational level.

To obtain approval for the study, information about the study and consent forms were sent to the heads of the healthcare sectors in seven healthcare districts in the two regions. After approval, information and invitations to participate in the questionnaire were sent to the head nurses for all 75 nursing homes in 19 municipalities in the two regions. The head nurses were requested to distribute the link to the questionnaire to all employees in the nursing staff by email. Due to General Data Protection Regulation (GDPR) [39], it was not possible to get the names of the participants and send the survey directly to them. All employees in the nursing homes were included in the data collection, and no exclusion criteria were mentioned. We also wanted to include staff without formal education and short-contracted employees. The online questionnaire was created using E-lomake software [40]. The expected time to answer the questionnaire was a maximum of 20 min and the participants could answer it during working time. The participants could choose to answer the questionnaire in either Swedish or Finnish. Several reminders were sent to the head nurse to encourage the participants. Additionally, hardcopy questionnaires were sent to nursing homes that did not use email or asked for hardcopy questionnaires and were returned in sealed envelopes. The data collection was conducted between December 2020 and January 2021. Power analysis [41] was conducted to determine the sample size of the study and a total of 126 participants should be included in the study sample to achieve a power of 80%, with p < 0.05. The effect size of Cohen’s *d* used in the analysis was 0.5, i.e., a medium size effect. However, RNs and PNs had score sheets where the correct answers differed from each other for some items; thus, the standard deviation in these subgroups was lower than in the whole group. Therefore, separate power analyses were also performed for both subgroups. Calculated with the same power and p-value assumptions as above, the sample size needed for both RNs and PNs was 126.

### Ethical considerations

The Research Integrity Advisers and the Board at Åbo Akademi University (FEN) gave approval for research ethics in April 2020. The study was conducted following the ethical principles of the Declaration of Helsinki [42] and the Finnish National Board of Research Integrity (TENK) [43]. Ethical considerations, such as confidentiality and voluntariness, were described in the information letter to the head nurses and their staff. In answering the questionnaire, approval was given to participate in the study. No personal information was asked in the questionnaire, only the municipality and the name of the nursing home and ward.

### Data analysis

Data analysis were carried out in IBM SPSS Statistics Version 26. The data from the questionnaire were transferred to SPSS from E-lomake [40] and the hardcopy questionnaires. A total of 351 participants answered the questionnaire and 14 participants with no relevant education or incomplete questionnaires were excluded from the study since their confidentiality could not be granted. Categorical variables were presented in frequencies and percentages and quantitative variables were presented in means and ranges for RNs and PNs, respectively. Missing data were not addressed, due to minimum missing data. Only completed questionnaires in the Ms. Olsen test was included in the data analysis. The missing data in the tables, i.e., gender and age, was so small and did not influence the results.

In the data analysis, a correct answer in the Ms. Olsen test was coded 1 and an incorrect answer was coded 0. The number of correct answers was 1 and/or 2, depending on the nursing staff’s profession (see Table 1). The participants were allowed to give two answers regarding Ms. Olsen’s symptoms. The correct option was chosen as their answer. The correct answer was coded 1 and the incorrect or missing answer was coded 0 in SPSS. The total variable score was calculated for each participant and 18 was the maximum points. The cut-off, minimal acceptable score for RNs is two-thirds (66.7%) of the total sum of their points and for PNs, half (50%) of the total sum [34]. In this test, the minimum acceptable scores, the cut-offs, were set at 12 and 9 for RNs and PNs, respectively.

Pearson’s chi-square test was performed to compare the differences between the groups of RNs and PNs in the proportion of correct answers to the top and bottom five items of the clinical competence test. The effect size, contingency coefficient (C), was also determined and was considered high when C > 0.40 and moderate when C was 0.25–0.4.

Spearman’s correlation was used to discover the relationship between the score of the nurses’ clinical competence and the background variables. The results were presented with a *p*-value and correlation coefficient (R). For all the statistical analyses, a *p*-value < 0.05 was considered statistically significant. For correlation analyses, dichotomisations were done for gender and mother tongue by excluding other and missing categories, respectively ownership, by combining the categories’ profit and non-profit to private. All significance tests were performed two-tailed.

## Results

A total of 337 participants were included in the study, of which 95 (28.0%) were RNs and 242 (72.0%) were PNs. A post hoc power analysis was also performed, and it determined a power of 1.00 for the total sample and the power for the professional groups was 0.68 for the RNs and 0.96 for PNs. The questionnaire was sent to the head nurse of the nursing home and we have no information on how many nursing staff received the email and the questionnaire. There were 18 participating municipalities in the two healthcare districts, two municipalities did not agree to participate and 16 participants did not mention the municipality in the questionnaire. There were 75 invited nursing homes and 50 (68%) nursing homes participated, while five participants did not mention the name of the nursing home. Based on the number of patients in the invited nursing homes, an estimated 1100 nursing staff were invited to participate. The theoretical minimum for the response rate was calculated to be 31%. The information about the number of patients was taken from the published information on the internet, and the number of nurses calculated was based on the legislation on nursing staff [32].

### Characteristics of participants

The majority of the participants were female (96.4%, *n* = 325), and the majority (57.3%, *n* = 193) had Swedish as their mother tongue, 37.7% (*n* = 127) were Finnish-speaking and only a minority (5.1%, *n* = 17) had English or another language as their mother tongue. More specific and detailed characteristics of the participants are provided in Table 2. English-speaking participants answered in Finnish or Swedish. The language chosen to answer was coded as their working language and coded into either Swedish 58.8% (*n* = 198) or Finnish 41.2% (*n* = 139). Only a few participants had English as their mother tongue.

The mean age of all participants was 43 years (range: 18–68). The mean age was 44 (range: 22–68) for the RNs and 42 (range: 18–67) for the PNs. The mean number of years of experience working in elderly care for all participants was 13.5 (range: 0–45). For the RNs, the mean number of working years in elderly care was 14.7 (range 0–45), and for the PNs, the mean was 13.0 (range 1–40). The mean number of working years in the actual nursing home was 7 (range 0–40), for the RNs 7.1 years (range: 0–25) and for the PNs 8.3 years (range: 0–38). The characteristics of the participants are presented in Table 2.

**Table 2:** Characteristics of participants. N = 337, RN = 95 & PN = 242

Variable		Count	%	RN Count	%	PN count	%
**Gender**	Female	325	96.4	90	94.7	235	97.1
	Male	10	3.0	4	4.2	6	2.5
	Missing& Other	2	0.3	1	1.1	1	0.4
**Education**	Masters in Nursing	10	2.8	10	10.5		
	Bachelor in Nursing	85	24.2	85	89.5		
	Bachelor in Social Work	6	1.7			6	2.5
	Practical nurse	231	65.8			231	95. 5
	Specialist vocational qualifications in elderly care	5	1.4			5	2.1
**Age**	18–30	81	24.0	19	20.0	62	25.6
	31–50	135	40.1	45	47.4	90	37.2
	51–60	103	30.6	24	25.3	79	32.6
	61–68	16	4.7	7	7.4	9	3.7
	Missing	2	0.6			2	0.8
**Work place**	Sheltered housing	90	26.7	24	25.3	66	27.3
	Institutional care	247	73.3	71	74.7	176	72.7
**Ownership**	Municipality	254	75.4	74	77.9	180	74.4
	Private	52	15.4	10	10.5	42	17.4
	Third sector	31	9.2	11	11.6	20	8.3
**Position**	Head Nurse or Leader	24	7.1	22	23.2	2	0.8
	RN	67	19.9	67	70.5		
	Social Worker	2	0.6			2	0.8
	PN	235	69.7	4	4.2	231	95.5
	Carer	8	2.4	2	2.1	6	2.5
	Care assistant	1	0.3			1	0.4
**Mother tongue**	Swedish	193	57.3	52	54.7	141	58.3
	Finnish	127	37.7	39	41.1	88	36.4
	Other	17	5.1	4	4.3	13	5.4
**Working language**	Swedish	198	58.8	52	54.7	146	60.3
	Finnish	139	41.2	43	45.3	96	39.7
**Position type**	Permanent	264	78.3	74	77.9	190	78.5
	Temporary	64	19.0	18	18.9	46	19.0
	Occasionally	9	2.7	3	3.2	6	2.5
**Position size**	up to 75%	92	27.3	18	18.9	74	30.6
	76–100%	242	71.8	77	81.1	165	68.2
	Missing	3	0.9			3	1.2

In the self-evaluation of clinical competence, the majority of the participants (86.4%, *n* = 291) reported that they had good to very good clinical competence, with 36.0% (*n* = 121) reporting that they had received continuing education in the workplace in the nursing field during the last year.

The Finnish Current Care Guidelines were used on a daily basis by 7.4% (*n* = 25) of the participants and weekly by 30% (*n* = 100). On a daily basis, 1.5% (*n* = 5) reported reading Swedish national guidelines and 3.9% (*n* = 13) reported using Google. The detailed descriptions of the questions regarding self-evaluated clinical competence, continuing education and acquisition of electronic healthcare information are shown in Table 3.

**Table 3:** Description of the questions regarding self-evaluated clinical competence, continuing education and acquisition of electronic health care information. N = 337; RN = 95 & PN = 242

Variable		Count	%	RN Count	%	PN Count	%
**Selfevaluation clinical competence**	Very good	93	27.6	35	36.8	58	24.0
	Good	198	58.8	51	53.7	147	60.7
	Pretty Good	42	12.5	9	9.5	33	13.6
	Weak	1	0.3			1	0.4
	Very weak						
	Missing	3	0.9			3	1.2
**Continuing education during worktime**	0 days/year	215	63.8	56	58.9	160	66.1
	1–3 days/year	98	29.1	32	33.7	65	26.9
	4–20 days/year	22	6.5	7	7.4	15	6.2
	> 60 days/year	1	0.3			1	0.4
	Missing	1	0.3			1	0.4
**Continuing education outside worktime**	Yes	40	11.9	12	12.6	28	11.6
	No	292	86.6	83	87.4	209	86.4
	Missing	5	1.5			5	2.1
**Finnish Current Care Guidelines**	Daily	25	7.4	6	6.3	19	7.9
	Weekly	100	29.7	41	43.2	59	24.4
	Monthly	158	46.9	42	44.2	116	47.9
	Never	54	16.0	6	6.3	48	19.8
**Swedish Current Care Guidelines**	Daily	5	1.5			5	2.1
	Weekly	40	11.9	6	6.3	34	14.0
	Monthly	91	27.0	28	29.5	63	26.0
	Never	201	59.6	61	64.2	140	57.9
**Google**	Daily	13	3.9	6	6.3	7	2.9
	Weekly	103	30.6	33	34.7	70	28.9
	Monthly	147	43.6	34	35.8	113	46.7
	Never	74	22.0	22	23.2	52	21.5

### Clinical competence

Among the RNs surveyed, 24.0% (*n* = 23) showed clinical competence with a score of 12 (cut-off for RNs) or higher, and 30.0% (*n* = 73) of the PNs had a score of 9 (cut-off for PNs) or higher. Table 4 shows the total scores and their distribution for the nursing staff and their clinical competence.

**Table 4:** Distribution of total score and cut off for accepted clinical competence for nursing staff in clinical competence. Cut off for RN = 12 and PN = 9. Horizontal lines represent cutoff levels for RN and PN.

RN = 95			PN = 242	
Score	*n*	%	*n*	%
0			3	1.2
1			6	2.5
2	2	2.1	11	4.5
3	2	2.1	16	6.6
4	2	2.1	27	11.2
5	4	4.2	23	9.5
6	8	8.4	35	14.5
7	12	12.6	22	9.1
8	11	11.6	26	10.7
9	11	11.6	21	8.7
10	12	12.6	13	5.4
11	8	8.4	17	7.0
12	7	7.4	10	4.1
13	8	8.4	7	2.9
14	4	4.2	2	0.8
15	1	1.1	2	0.8
16	2	2.1	1	0.4
17				
18	1	1.1		
Above cut off	23	24.2	73	30.2
Total	95	100.0	242	100.0

### Top and bottom items for clinical competence

The five items showing the best clinical competence are presented in Table 5. The lowest clinical competence for the items is presented in Table 6; both the RNs and PNs had the lowest competence in tiredness and keeping the room organised.

**Table 5:** The five items showing best clinical competence. The number of RN = 95 and PN = 242

Rank	RN	*n*	%	PN	*n*	%
1	Is substantially dehydrated	86	90.5	Has newly occurring chest pain	207	85.5
2	Choughs. has increased saliva and respiration frequency above 20/min	83	87.4	Has symptoms of partial paralysis	191	78.9
3	Has fresh blood in stool	72	75.8	Has fresh blood in stool	176	72.7
4	Has symptoms of partial paralysis	68	71.6	Is substantially dehydrated	128	52.9
5	Has dyspnoea during rest within last two days	65	68.4	Has dyspnoea during rest within last two days	120	49.6

**Table 6:** The five items showing lowest clinical competence. The number of RN = 95 and PN = 242

Rank	RN	*n*	%	PN	*n*	%
1	Is more tired during the day	8	8.4	Is more tired during the day	9	3.7
2	Has lost interest in keeping home in order. sleeps in chair instead of bed	9	9.5	Has lost interest in keeping home in order. sleeps in chair instead of bed	18	7.4
3	Has pain and discomfort in mouth	15	15.8	Choughs. has increased saliva and respiration frequency above 20/min	39	16.1
4	Has changes in sight, hearing, speech and comprehension	25	26.3	Has fallen two times during previous week	42	17.4
5	Skin has rash, wounds, is red or itchy	29	30.5	Has pain and discomfort in mouth	44	18.2
5	Has reduced appetite and food intake	29	30.5			

The Pearson’s chi-square test detected significant differences (p < 0.05) between the two groups, the RNs and PNs, in 10 of the 18 items (see Table 7). The biggest difference between the groups, presented as effect size, was for the items *Coughs, has increased saliva and respiration frequency above 20/min* (C = 0.55) with a big effect and *Has fallen two times during previous week* (C = 0.38) with a moderate effect.

**Table 7:** Description of the count and percentages and significant differences (p < 0.05) between the groups for the items for clinical competence. RN = 95 and PN = 242

Item wording statement	Count correct RN	%	Count correct PN	%	*p*	Contingency coefficient*
Has dyspnoea during rest within last two days	65	68.4%	120	49.6%	0.002	0.168
Choughs, has increased saliva and respiration frequency above 20/min	83	87.4%	39	16.1%	0.000	0.555
Has temperature above 38.5	57	60.0%	98	40.5%	0.001	0.173
Is substantially dehydrated	86	90.5%	128	52.9%	0.000	0.332
Skin has rash, wounds, is red or itchy	29	30.5%	73	30.2%	0.948	0.004
Has reduced appetite and food intake	29	30.5%	100	41.3%	0.067	0.099
Is not able to eat	37	38.9%	87	36.0%	0.608	0.028
Has pain and discomfort in mouth	15	15.8%	44	18.2%	0.603	0.028
Is incontinent for urine, stings when urinates	57	60.0%	76	31.4%	0.000	0.255
Has fresh blood in stool	72	75.8%	176	72.7%	0.566	0.031
Has increased needs to full care within last two days	60	63.2%	54	22.3%	0.000	0.362
Has fallen two times during previous week	56	58.9%	42	17.4%	0.000	0.381
Has symptoms of partial paralysis	68	71.6%	191	78.9%	0.150	0.078
Is more tired during the day	8	8.4%	9	3.7%	0.076	0.096
Has changes in sight. hearing, speech and comprehension	25	26.3%	96	39.7%	0.021	0.124
Has newly occurring chest pain	64	67.4%	207	85.5%	0.000	0.202
Has lost interest in keeping home in order, sleeps in chair instead of bed	9	9.5%	18	7.4%	0.536	0.034
Has short attention span and delusions	50	52.6%	96	39.7%	0.031	0.117

*Contingency coefficient is measuring the effect size

### Correlations between scores for clinical competence and background variables

Spearman’s rank correlations between the scores for clinical competence and background variables are presented in Table 8. A statistically significantly moderate correlation was found for the nursing staff, Swedish as working language (*r* = 0.395; *p* = 0.000), mother tongue (*r* = 0.331; *p* = 0.000) and the score for clinical competence. Statistically significantly weak correlations were found between the scores for clinical competence and age (*r* = 0.187; *p* = 0.001), years in elderly care (*r* = 0.118; *p* = 0.031), continuing education outside work (*r* = -0.059; *p* = 0.016) and using the Swedish national guidelines (*r* = 0.194; *p* = 0.000). No statistically significant correlation was found between the nursing staff’s clinical competence using the Finnish Current Care Guidelines (*r* = -0.009; *p* = 0.871).

**Table 8:** Correlations between clinical competence and background variables

Background variable	Clinical competence¹¹ Total (*n* = 337)		Clinical competence¹¹ RN (*n* = 95)		Clinical competence¹¹ PN (*n* = 242)	
	Correlation coefficient	*p*	Correlation coefficient	*p*	Correlation coefficient	*p*
Gender¹	0.034	0.540	0.120	0.249	-0.011	0.870
Age in year	0.187^**^	0.001	0.207^*^	0.044	0.178^**^	0.006
Education²	-0.067	0.217	0.046	0.656	-0.055	0.393
Profession³	-0.066	0.231	-0.050	0.631	-0.018	0.783
Work place^4^	0.039	0.473	0.102	0.102	0.018	0.776
Ownership^5^	-0.086	0.115	-0.140	0.178	-0.056	0.388
Position type^6^	0.057	0.295	-0.056	0.591	0.099	0.125
Position size in %	-0.080	0.146	-0.037	0.722	-0.079	0.225
Years in elderly care	0.118^*^	0.031	0.205^*^	0.046	0.074	0.252
Years in current work place	0.104	0.058	0.087	0.404	0.104	0.109
Mother tongue^7^	0.331^**^	0.000	0.350^**^	0.001	0.322^**^	0.000
Working language^8^	0.395^**^	0.000	0.415^**^	0.000	0.386^**^	0.000
Selfevaluation of clinical competence^9^	0.005	0.928	0.218^*^	0.034	-0.066	0.313
Continuing education during worktime (days/year)	-0.059	0.281	-0.008	0.942	-0.077	0.236
Continuing education outside work (days/year)	-0.132^*^	0.016	0.007	0.946	-0.185^**^	0.004
Finnish Current Care Guidelines^10^	-0.078	0.154	-0.097	0.351	-0.063	0.328
Swedish Current Care Guidelines^10^	0.194^**^	0.000	0.330^**^	0.001	0.143^*^	0.026
Google^10^	-0.085	0.118	-0.206^*^	0.045	-0.032	0.624

* Correlation is significant at the 0.05 level (2-tailed)

** Correlation is significant at the 0.01 level (2-tailed)

Classification of the categories on ordinal level:

¹Gender: 1 = Male; 2 = Female

²Education: 1 = Practical Nurse; 2 = Specialist vocational in elderly care; 3 = Bachelor in Social work; 4 = Bachelor in Nursing; 5 = Master in Nursing

³Profession: 1 = Career; 2 = PN; 3 = Social worker; 4 = RN; 5 = Head or Leader

^4^Work place: 1 = Sheltered housing; 2 = Institutional care

^5^Ownership: 1 = Municipality; 2 = Private (for profit and non-profit)

^6^Position type: 1 = Occasionally; 2 = Temporary; 3 = Permanent

^7^Mother tongue: 1 = Finnish 2 = Swedish

^8^Working language: 1 = Finnish; 2 = Swedish

^9^Self-evaluation of clinical competence: 1 = Really weak; 2 = Weak Really; 3 = Pretty good; 4 = Good; 5 = Really good;

^10^National guidelines & Google: 1 = Never; 2 = Every month; 3 = Every week; 4 = Every day

¹¹Clincal competence: 1 = Has clinical competence; 0 = No clinical competence

## Discussion

The aging population in Finland is growing, resulting in a higher prevalence of diseases and longer lifespans, leading to an increased demand for nursing home facilities. Unfortunately, investigations conducted by Finnish authorities have revealed inadequate nursing care due to insufficient staffing and competency among personnel. Therefore, the aim of this study was to explore, for the first time in Finland, the RNs’ and PNs’ clinical competence and decision-making skills in nursing homes for older people in Finland and to analyse the association between nurses’ clinical competence and background factors.

The main findings of this study were the worrisome limitations in the clinical competence of many nursing staff members in LTC in the western part of Finland. The Ms. Olsen test revealed that only one-fourth of the RNs and a third of the PNs passed the cut-off for the clinical competence test. However, the nursing staff evaluated their own clinical competence to be high, with over 80% evaluating regarded themselves as having good or very good clinical competence. More than half of the nursing staff admitted not having participated in any continuing education during working time during the last year before the data collection.

Our research stated that clinical competence was relatively low for both RNs and PNs in Finland. This is in line with the findings of the Norwegian study, where the level of clinical competence was found to be insufficient and the RNs were more competent than the PNs [6, 44]. Finnish researcher Kiljunen [13] has stated that RNs need to have high competence because they supervise nursing staff with a lower level of education. Tohmola et al. [45] confirmed that clinical competence was needed in the Finnish LTC since the nursing staff needed to detect changes in the older people’s health status to provide the correct nursing care. A Taiwanese study indicated that nurses had a moderate level of clinical competence and the score for interpersonal relationships was the highest, followed by clinical competence in disease and nursing skills [46]. Competence is needed to provide advanced medical treatments and nursing care to older patients, who are more complex individuals with multifaceted conditions and diseases [47].

Almost all (98.9%) participants evaluated themselves as having *pretty good* to *very good* clinical competence. This is surprising since the results showed that only a fourth of the RNs and one-third of the PNs passed the clinical competence test. The expertise of nursing staff was previously examined in home care in Finland with the help of the Nurse Competence in Care Home Scale (NCCHS) self-assessment tool [19]. In a Finnish study [20], the participants evaluated their own clinical competence in the care of patients with dementia to be at a moderate level with a positive correlation with age, work experience with people with dementia and work experience in the current ward.

In Finland, and especially in bilingual regions, patients have the right to receive nursing care in their mother tongue since the patients speak either Swedish or Finnish, meaning there is both Finnish and Swedish nursing education. Our study was conducted in a bilingual region, and among the nursing staff who participated, we found that those with Swedish as their mother tongue or working language had, on average, higher clinical competence. For the RNs and their scores for clinical competence, a statistically significant moderate correlation was found between clinical competence and Swedish as a working language, their mother tongue and a moderate correlation for using Swedish Current Care Guidelines; The Handbook of Healthcare [48] and Internetmedicin [49]. For the PNs, a significantly moderate correlation was found between Swedish as the mother tongue and working language with clinical competence. This study showed that Swedish-speaking nursing staff who also used the Swedish Current Care Guidelines [48, 49] had higher clinical competence. Based on this study, it is not possible to state any possible reasons for this finding. To the best of our knowledge, this is the first study showing differences in clinical competence between Swedish- and Finnish-speaking nursing staff in nursing homes in bilingual regions.

Previous studies have shown that the average age of nursing staff in LTC is relatively high [50–52]. This was similar in our study, in which the mean age of all the participants was relatively high and a third of the PNs were aged between 51 and 60, which shows that the PNs’ age was rather high. Our study showed a weak correlation between clinical competence and the age of the nursing staff. The RNs had a significant correlation between clinical competence and age, but the PNs had a significantly weak correlation between clinical competence and age. In Norway, age had a negative impact on clinical competence, where older nursing staff scored lower than younger nursing staff [6]. However, our study does not show the reason for this and further investigations needs to be done.

The number of years working in elderly care showed a significant correlation with clinical competence in the total sample, which is in line with earlier studies [51, 52]. However, we found significant correlations between clinical competence and working years for RNs but not for PNs. We found that the longer the working experience in elderly care, the better the likelihood of higher clinical competence, as measured by the Ms. Olsen test. The nursing staff’s average working experience was long, with an average of 13 years working in elderly care and 8 years working in the current nursing home. This is in line with another Finnish study, which stated that age and work experience in the current department have positive correlations with clinical competence, especially for RNs [19, 20]. However, interestingly, other studies have shown that age and work experience are not predictors of clinical competence [6, 52]. Benner [9] has stated that after 2–3 years of work experience, a nurse is competent. With an average of 13 working years in our study, the nurses should either be at a proficient or expert level; however, our study showed that the nurses had relatively low clinical competence, but further research needs to be done for further understanding.

Studies have shown that the higher the education level [53], the better the nursing staffs´ clinical competence. However, our research did not show that participants with a master’s level had better clinical competence. Moreover, in Finland, nursing education at a master’s level is more theoretical than clinical oriented and could therefore explain this finding and further research need so to be done.

The majority of our participants had a permanent position and other research has proven that nursing staff with a permanent position was a positive aspect for clinical competence [46]. Thus, we did not find any significant correlation between the type and size of position in our research, nor if the nursing staff was employed by the municipality, private or the third sector. Nursing care in Finland has a lack of nursing staff and the policies for the employees are safe, which may indicate that nursing staff do not need to get permanent positions or compete for their positions.

Continuous education plays a central role in LTC. Our research showed that continuous education was not prioritised; only 12% of all participants replied that they had participated in continuing education in the nursing field in either their working time or spare time. We must also bear in mind that this research was done during the COVID-19 pandemic, when further education was cancelled in nursing homes, but they could have participated before the pandemic started. Other research has shown that continuous education is central to improving clinical competence among nursing staff [47, 52], especially PNs [19]. The participants in Finnbakk et al.’s study revealed that continuous education would give nursing staff more inspiration and a higher status to work in LTC and train them to perform clinical assessments and decision-making for complex older patients [47].

Only a minority of the participants reported using the Finnish Current Care Guidelines [24] on a daily basis, a third on a weekly basis and almost a fourth reported they have never used it. The latest guidelines for correct nursing care are collated on the platform [24], and these should be used by the nursing staff. According to evidence-based practices, providing high-quality nursing care is a requirement and it is the nurses’ responsibility to verify the latest care guidelines for the patient [17]. The result was low, with only one-third of the nursing staff verifying the Finnish Current Care Guidelines [24] on a weekly basis. The Swedish-speaking nursing staff also verified the Swedish databases, The Handbook of Healthcare [48] and Internetmedicin [49], probably because Swedish is easier to read and understand for them. Here, we must note that the guidelines can differ for each country.

The Ms. Olsen test showed that RNs’ best clinical competencies were to recognise and act for dehydration and breathing difficulties and for the PNs to recognise and act for chest pain and partial paralysis. However, the groups cannot be compared since both RNs and PNs have their own formal clinical competencies. The groups had different correct answers on the score sheet and these are related to their clinical competence and education. Both groups’ lowest competence was to recognise and act for fatigue and to lose interest in their surroundings. Oral problems had a low result in both groups and this was in line with other research that implicated that oral care was not prioritised in nursing homes, though it is part of nursing responsibilities, but can cause difficulties and ethical dilemmas in performing oral care [54]. The bottom five items can be seen as a gap in the nursing staff’s clinical competence and therefore an indication that continuing education should be provided to the nursing staff to help them improve their assessment in subjective measures of symptoms, such as tiredness, cognitive assessment and mouth care. This may indicate that the nursing staff were neglecting these symptoms in the patients, which could have negatively affected the quality of LTC.

### Limitations

The response rate was calculated on a theoretical level. Due to the GDPR, we could not reach the total sample in person, and we had to rely on the managers’ help to encourage the participants. Therefore, we did not have detailed information about the total sample. However, there was good communication with the managers in the regions who forwarded the questionnaires to the total sample. Due to the COVID-19 pandemic, data collection could only be done online and not in person. This might have affected the participants’ interest in taking part in the study. However, we received replies from a sufficient number of participants, and we were able to complete the study. The results of this study cannot be generalized because of the limited information about the total sample and the first testing of this study in Finland. This will, however, give us a good foundation for further research to develop the instrument.

The sample size was calculated for parametric tests. However, a nonparametric Spearman’s rank correlation was used since the Pearson correlation was not suitable for part of background variables. As a nonparametric test, Spearman correlation is slightly weaker than Pearson correlation, but still fully possible to use because the sample size was remarkably larger than the power calculation would have required. In this study, analyses for the different professional subgroups, RNs and PNs, were also performed and only in the subgroup of RNs was the sample size left below the target. However, this will provide new knowledge for the development of the instrument.

The data collection was done with the nursing staff in the western part of Finland, with more Swedish speaking nursing staff than Finnish speaking, which means it does not cover the total sample of the nursing staff in Finland, but it is a good starting point for further research. Both the Swedish and Finnish questionnaires have proven to be equally good and were used in a geographical area where both languages are utilized. Although surprising, the results indicate that the significance of language as a contextual factor that impacts nursing care cannot be overlooked. Nevertheless, additional research is needed to explore the bilingual setting and determine how the development of clinical competence may affect it.

One of the 19 items was removed due to interpretation errors. The cut-off level was calculated in percentages and was the same for both 18 and 19 items; therefore, this did not affect the results.

## Conclusion

For the first time in Finland, a clinical competence test is done with the Norwegian Ms. Olsen test and the level of the nursing staffs’ clinical competence in long-term care has evaluated and mapped. We found that, according to the Ms. Olsen test, nursing staff showed concerning limitations in their clinical competence in Finnish nursing homes, both for RNs and PNs. Only 24% of the RNs and 30% of the PNs passed the Ms. Olsen test. These results differed remarkably from their self-assessments, in which the majority of the nursing staff found their competence to be good or very good. We also found that the nursing staff did not use the Finnish Current Care Guidelines as often as they should to develop and update their own nursing skills and knowledge.

In addition, we identified a competence gap among the nursing staff, particularly concerning limited clinical competence in the specific items, *Is more tired during the day* and *Has lost interest in keeping home in order, sleeps in chair instead of bed*. A competence gap identified through an instrument, such as the Ms. Olsen test, can be used to develop targeted continuous education.

### Implications for further research

Further research is needed regarding nursing care related to evidence-based practice. A gap seem to appear between the education and the current nursing work, as is it based on old routines and traditions and the nursing staff need to be encouraged more to develop their nursing competence. Further research is needed to understand the predictors of clinical competence in LTC and at what level stress, culture and routines affect clinical competence and missed care. More research is needed in bilingual nursing context to see whether there is an effect of the nursing staffs’ development of clinical competence.

### Implications for nursing practice

Our research shows the current status, that of a concerning limited clinical competence, of nursing staff in elderly care in Finland. A gap in the nursing staff’s clinical competence has been identified and targeted continuous education can be developed to secure decision-making skills, high quality and patient safety in elderly care.

## Data Availability

The datasets generated and analysed during the current study are not available for public use, due to confidentiality, but are available from the corresponding author on reasonable request.

## Abbreviations

C: Contingency coefficient
LTC: Long-term care
*M*: Mean
*n*: Sample size
*p*: Probability value
NCCHS: Nurse Competence in Care Home Scale
NOP-CET: Nursing Older People-Competence Evaluation Tool
PN: Practical nurse
RN: Registered nurse
STROBE: Strengthening the Reporting of Observational studies in Epidemiology.
